# A Female-Biased Odorant Receptor from *Apolygus lucorum* (Meyer-Dür) Tuned to Some Plant Odors

**DOI:** 10.3390/ijms17081165

**Published:** 2016-07-28

**Authors:** Zhixiang Zhang, Meiping Zhang, Shuwei Yan, Guirong Wang, Yang Liu

**Affiliations:** 1 School of Life Science, Shanxi Normal University, Linfen 041000, China; zhang3121414@126.com; 2 State Key Laboratory for Biology of Plant Diseases and Insect Pests, Institute of Plant Protection, Chinese Academy of Agricultural Sciences, Beijing 100193, China; ysw_0220@163.com (S.Y.); grwang@ippcaas.cn (G.W.)

**Keywords:** *Apolygus lucorum*, odorant receptor, plant volatile, *Xenopus* oocytes, electroantennogram

## Abstract

*Apolygus lucorum* (Meyer-Dür) (Hemiptera: Miridae) is a serious pest of cotton, jujube, grape and many other crops around the world. Understanding how olfactory information directs this insect to its host plants may provide environment-friendly approaches to the control of its population in agriculture. In our study, we cloned an odorant receptor gene, *AlucOR46*, that was specifically expressed in antennae and female-biased. Functional expression of AlucOR46 in *Xenopus* oocytes showed that it is tuned to six plant volatiles (*S*)-(−)-Limonene, (*R*)-(+)-Limonene, (*E*)-2-Hexenal, (*E*)-3-Hexenol, 1-Heptanol and (*1R*)-(−)-Myrtenol. Electroantennogram (EAG) recordings revealed that all six compounds could elicit electrophysiological responses from the antennae of *A. lucorum*, higher in females. Our results are in agreement with previous reports showing that (*E*)-2-Hexenal could attract female *A. lucorum* in behavior experiments. These results suggest that AlucOR46 might play an important role in locating the host plants of *A. lucorum* and therefore represents a suitable target for green pest control.

## 1. Introduction

The green mirid bug, *Apolygus lucorum* (Meyer-Dür) (Hemiptera: Miridae) is an important worldwide pest. Before commercial cultivation of transgenic *Bacillus thuringiensis* (Bt) cotton in China, *Helicoverpa armigera* was the major cotton pest while populations of *A. lucorum* were kept at a low level. However, since the widespread planting of trans-Bt cotton and reduction of insecticide spraying, the population of *A. lucorum* increased dramatically so that it has become the key insect pest for cotton and several other agricultural crops [1]. Due to its serious damage in agriculture, *A. lucorum* became the object of many studies on its life cycle, habits and host plants preference [2,3,4]. *A. lucorum* is a highly polyphagous insect and feeds on over one hundred plant species. Furthermore, it can easily and frequently switch between habitats and host plants [1,3,5]. As shown in previous studies, host preference and other behaviors in insects are modulated by host plant volatiles [6,7]. Therefore, accurate perception of plant volatiles is highly important for the adaptability of this phytophagous insect to different environmental conditions. Behavior experiments using a Y-shaped olfactometer in the laboratory and field observations were performed to study the preferences of *A. lucorum* adults to six different host plant species and their volatiles [8]. Electroantennogram (EAG) responses of adult *A. lucorum* to different plant volatiles have also been recorded [9]. Six electrophysiologically active compounds were identified, four of which also proved to be strong attractants for adults of *A. lucorum* [10]. However, the physiological and biochemical mechanisms underlying the detection of these compounds still need to be investigated.

A sophisticated olfactory system is essential for survival and reproduction, allowing insects to locate food sources, identify mates and avoid predators [11]. Odorants entering olfactory sensilla interact with soluble odorant-binding proteins (OBPs), present in the sensillar lymph, and with membrane-bound odorant receptors (ORs) [12]. ORs, which represent the key elements of odor detection, present seven transmembrane domains (TMDs) with inverted membrane topology, where the N-terminus is intracellular and the C-terminus extracellular [13,14,15]. They are believed to act as ligand-gated ion channels formed by the interaction of individual specific receptors with a conserved co-receptor, named Orco [15,16,17,18]. RNA interference (RNAi) experiments have shown that Orco is required for olfaction in *A. lucorum* [19]. In our previous study, we have cloned four OR genes from this species, one of which was tuned to (*Z*)-3-Hexenyl acetate and several other floral compounds [20]. 

In the present study we report the functional characterization of another OR (*AlucOR46*), selected among the 80 OR genes identified in the antennae by a transcriptome project (Yang Liu, unpublished), selected on the basis of its female-biased expression. When expressed in *Xenopus* oocytes, AlucOR46 specifically responds to six plant volatiles that also elicit electrophysiological responses from the antennae of *A. lucorum* in a female-biased fashion. This receptor might be involved in locating the host plant and could represent a candidate target for pest control.

## 2. Results

### 2.1. Gene Cloning and Sequence Analysis

**Figure 1:**
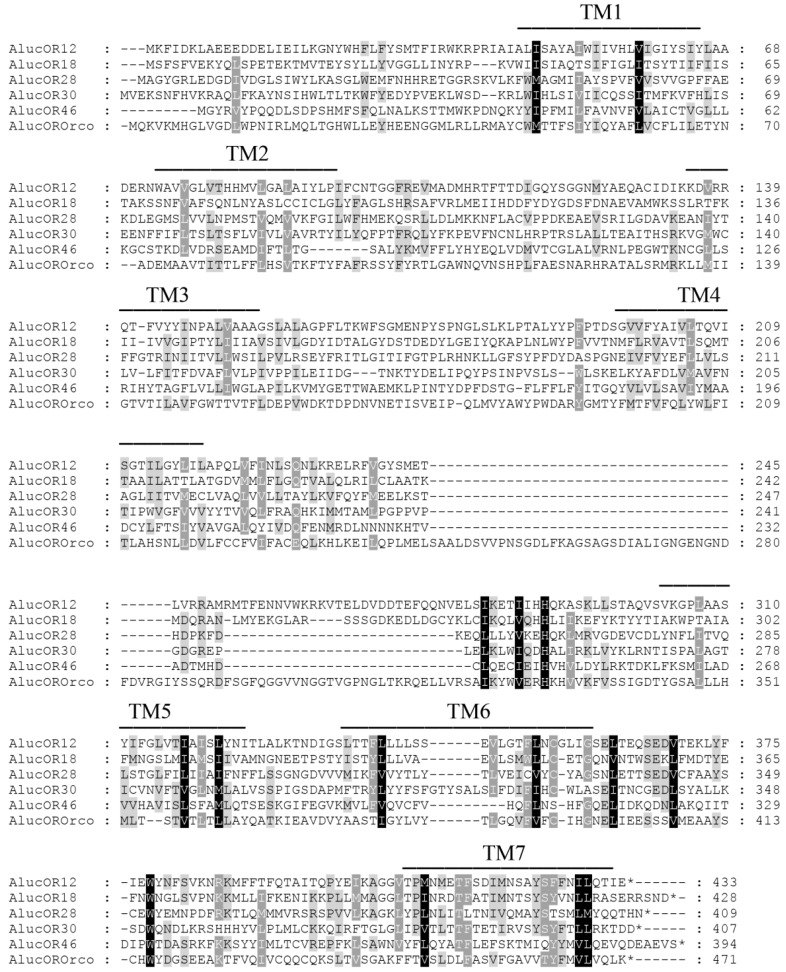
Alignment of amino acid sequences of AlucOR46, AlucOR12, AlucOR18, AlucOR30, AlucOR28 and AlucOrco. The seven transmembrane domains (TM1–TM7) are marked by solid lines. The conserved amino acid sites among the 6 ORs are marked with black shading. Amino acid similarities are very poor between these members. In particular, AlucOR46 is 12.7% identical to AlucOrco and shares 15.7%, 13.7%, 14.2% and 10.4% amino acids with AlucOR12, AlucOR18, AlucOR28 and AlucOR30, respectively.

Based on the analysis of *A. lucorum* antennal transcriptome (Yang Liu, unpublished), we cloned a full-length OR gene, *AlucOR46* (GenBank accession number: KU188516), with an open reading frame (ORF) of 1185 bp, encoding a protein of 394 amino acids. AlucOR46 is predicted to present 7 TMDs with an intracellular N-terminus and an extracellular C-terminus. Its identity with other ORs of the same species is very poor, 12.7% with AlucOrco and between 10% and 16% with other individual ORs (Figure 1).

### 2.2. Tissue Expression Pattern of AlucOR46

**Figure 2:**
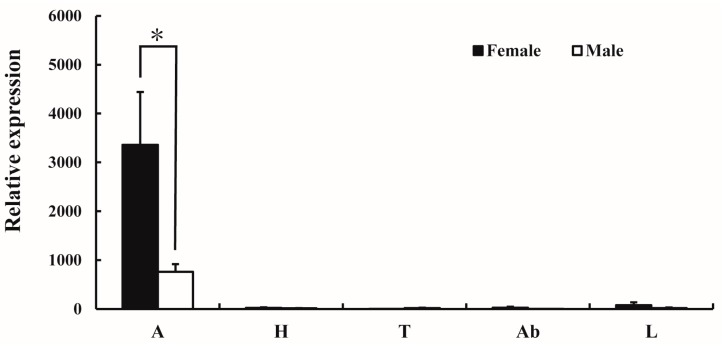
Tissue expression patterns of AlucOR46 in adults of *A. lucorum*. A: antenna; H: heads without antenna; T: thoraxes; Ab: abdomens; L: legs. Asterisk indicates significant difference between female and male.

The expression pattern of *AlucOR46* in different tissues of male and female *A. lucorum* was monitored by quantitative real-time PCR (qRT-PCR). The results show that *AlucOR46* was mainly expressed in antennae with female levels nearly four times those of males, making *AlucOR46* a typical female-biased gene (Figure 2). 

### 2.3. Functional Characterization of AlucOR46

**Figure 3:**
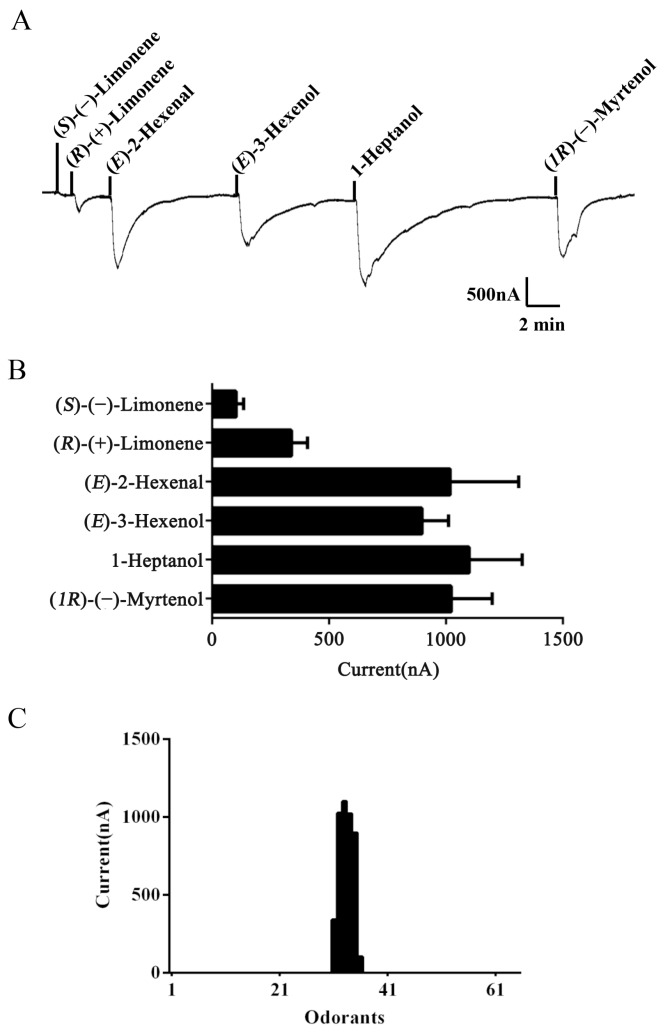
Functional characterization of AlucOR46/Orco in *Xenopus* oocytes. (**A**) Inward current responses of AlucOR46/Orco *Xenopus* oocytes to 10^−4^ M solution of (*S*)-(−)-Limonene, (*R*)-(+)-Limonene, (*E*)-2-Hexenal, (*E*)-3-Hexenol, 1-Heptanol and (*1R*)-(−)-Myrtenol; (**B**) Response profile of AlucOR46/Orco *Xenopus* oocytes. Error bars indicate standard error of the mean (SEM) (*n* = 6); (**C**) Tuning curve of AlucOR46. Tuning curve for the AlucOR46 to an odor panel comprising 65 odorants arranged along the *x*-axis. The odors which elicited the strongest responses are in the middle of the distribution, the weakest near the edges.

**Figure 4:**
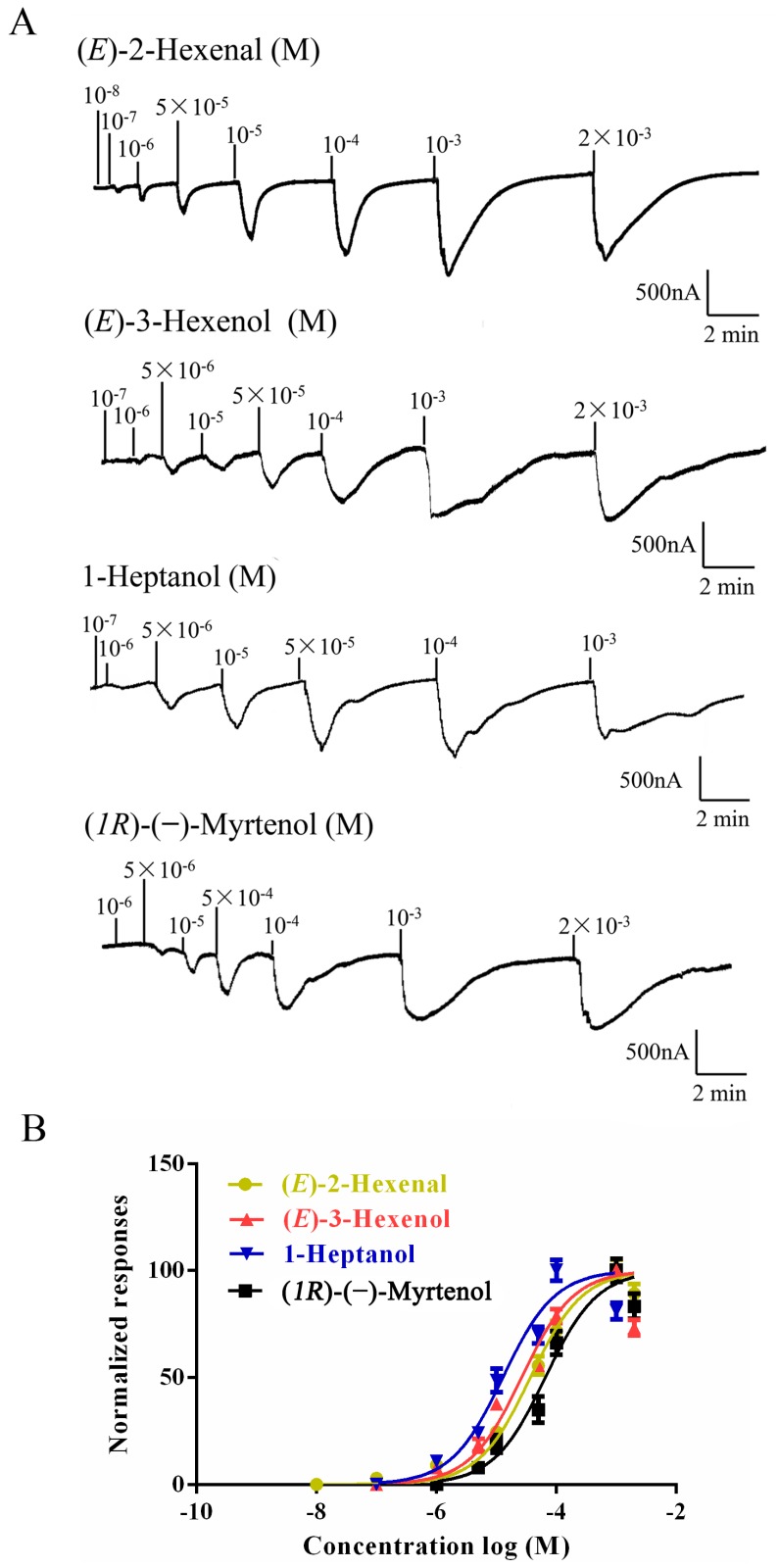
Dose-response of AlucOR46/Orco expressed in *Xenopus*. (**A**) AlucOR46/Orco *Xenopus* oocytes were stimulated with a concentrations range of (*E*)-2-Hexenal, (*E*)-3-Hexenol, 1-Heptanol and (*1R*)-(−)-Myrtenol; (**B**) Dose-response curves of AlucOR46/Orco *Xenopus* oocytes to (*E*)-2-Hexenal, (*E*)-3-Hexenol, 1-Heptanol and (*1R*)-(−)-Myrtenol. Responses are normalized by defining the maximal response as 100 in each group. The Error bar indicates SEM (*n* = 6).

To identify ligands for this receptor, *AlucOR46* was coexpressed with *AlucOrco* in *Xenopus* oocytes and responses to odorants were recorded using two-electrode voltage clamp. We used 65 compounds including terpenoids, alcohols, aldehydes and benzoates. Only six compounds: (*S*)-(−)-Limonene, (*R*)-(+)-Limonene, (*E*)-2-Hexenal, (*E*)-3-Hexenol, 1-Heptanol and (*1R*)-(−)-Myrtenol were found to elicit responses from AlucOR46/Orco, the last four being stronger than the other two (Figure 3). We then determined dose-response curves for the best four compounds and calculated half maximal effective concentration (EC_50_) values to be 3.71 × 10^−5^, 2.53 × 10^−5^, 1.38 × 10^−5^ and 7.44 × 10^−5^ M for (*E*)-2-Hexenal, (*E*)-3-Hexenol, 1-Heptanol and (*1R*)-(−)-Myrtenol, respectively (Figure 4 and Table 1).

### 2.4. Electrophysiological Responses of A. lucorum Antennae

**Figure 5:**
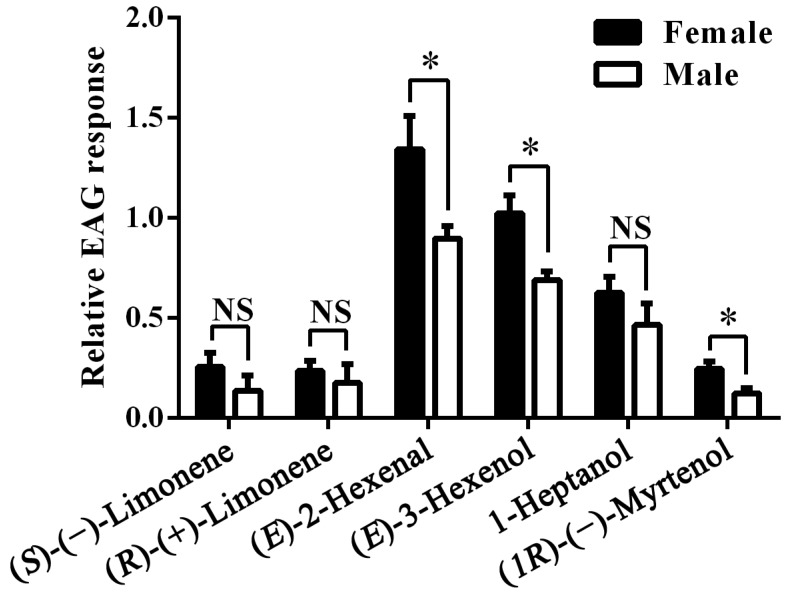
Relative electroantennogram (EAG) responses of female and male *A. lucorum* to six plant volatiles. NS indicates that there are no significant differences. Asterisks indicate significant differences in EAG response between female and male antennae, *p* < 0.05. Error bars indicate SEM (*n* = 6).

Next we recorded the electrophysiological responses of female and male antennae of *A.* *lucorum* to the six compounds selected in the *Xenopus* assay. The mean EAG response value to the blank stimulus (10 μL hexane) was 84.44 ± 3.74 (mean ± SEM) μV for *A. lucorum* females and 80.28 ± 4.84 μV for males. The mean response values to the reference stimulus (10 μL of 0.1 M 1-Hexanol), was 219.14 ± 14.46 μV for females and 222.22 ± 15.86 μV for males, which were significantly higher than to the blank stimulus (both *p* < 0.001). The difference between females and males in their responses to the blank or reference stimulus was not significant (*p* = 0.274 and 0.448). (*E*)-2-Hexenal and (*E*)-3-Hexenol proved to be the strongest stimuli, followed by 1-Heptanol. The other three stimuli produced much weaker signals. Responses were generally similar between sexes, in some cases significantly higher in female antennae (Figure 5).

## 3. Discussion

The selective response of AlucOR46 to plant volatiles and its female-biased expression suggest that this receptor could be involved by *A. lucorum* in locating host plant for feeding and oviposition [21,22,23].

In several studies, functional characterization of insects’ OR has been successfully conducted using heterologous systems [24,25,26,27]. When expressed in *Xenopus* oocytes, AlucOR46 was tuned to six plant volatiles, with stronger responses and higher sensitivity to (*E*)-2-Hexenal, (*E*)-3-Hexenol, 1-Heptanol and (*1R*)-(−)-Myrtenol. Except for (*1R*)-(−)-Myrtenol, the other three chemicals are all straight chain compounds and are similar in structure (Table 1). Different chemicals with similar structures could activate the same OR, a fact proven by many in vitro experiments. For example, in *Spodoptera exigua*, SexiOR3 was narrowly tuned to *E*-β-farnesene and several compounds of related structure [28]. However, although the four chemicals could stimulate similar responses in heterologous expression, the EAG responses to them in both female and male showed significant differences. This may be caused by the fact that olfactory selectivity does not only depend on ORs but also on other olfactory genes such as OBPs, sensory neuron membrane proteins (SNMPs) and odorant-degrading enzymes (ODEs), as well as from the expression level of these genes [12]. There is also a possibility that other ORs tuned to the same compounds may exist in the olfactory system of this species.

Previous studies in other mirids showed differences in the EAG responses between sexes. Males were more sensitive to insect-produced pheromones, while females were more sensitive to plant volatiles, cues to oviposition sites [29,30]. The EAG results of (*E*)-2-Hexenal and 1-Heptanol in our study are consistent with this report; however, the other four compounds were not mentioned in previous studies. Specifically, in both *Lygus lineolaris* and *Lygocoris pabulinus*, female antennae produced larger EAG responses than male’s to both the above compounds, with (*E*)-2-Hexenal eliciting stronger responses than 1-Heptanol in both sexes [29,30].

(*E*)-2-Hexenal is one of the green leaf volatiles (GLVs) released by plants after mechanical damage or herbivores attack. Herbivores use GLVs to locate host plants and plants also use GLVs to attract predators of pests to protect themselves [31,32,33]. Previous studies in Y-tube olfactometer and field trapping showed that *A.* *lucorum* females, but not males, could be significantly attracted by (*E*)-2-Hexenal, (*Z*)-3-Hexenol, phenylacetaldehyde and acetophenone, which are among the volatiles of cotton, alfalfa and cowpea [34]. Our results suggest that (*E*)-2-Hexenal might play an important role in locating host plants for females of *A.* *lucorum*. Besides, insects could also generate this compound. For example, *L. lineolaris* releases high doses of (*E*)-2-Hexenal when it is disturbed. This chemical is absent in the volatiles collected from calm males [35]. 

In conclusion, AlucOR46 is a receptor tuned to host plant volatiles and could represent an attractive target to control this important agricultural pest.

## 4. Materials and Methods 

### 4.1. Insect Rearing and Tissue Collection

The *A. lucorum* (Meyer-Dür) used in all experiments were obtained from a laboratory colony established and maintained at the Institute of Plant Protection, Chinese Academy of Agricultural Sciences, Beijing, China. Insects were reared with fresh corns and green beans and maintained at 28 ± 1 °C, with 60% ± 5% relative humidity and a 14 h:10 h light:dark photoperiod. Antennae, heads (without antennae), thoraxes, abdomens and legs were collected from male and female adults on the third day after eclosion, immediately frozen in liquid nitrogen and stored at −70 °C.

### 4.2. Plant Volatile Compounds

The 65 odorants tested in this study are listed in Table S1. All the chemicals were purchased from Sigma-Aldrich (Saint Louis, MO, USA). In two-electrode voltage-clamp electrophysiological recordings, odorants were dissolved in dimethyl sulphoxide (DMSO) as 1 M stock solutions. Before experiments, these were diluted to the appropriate concentrations in 1× Ringer’s buffer (96 mM NaCl, 2 mM KCl, 5 mM MgCl_2_, 0.8 mM CaCl_2_, and 5 mM HEPES, pH 7.6). In EAG experiments, the six selected compounds, (*S*)-(−)-Limonene, (*R*)-(+)-Limonene, (*E*)-2-Hexenal, (*E*)-3-Hexenol, 1-Heptanol and (*1R*)-(−)-Myrtenol, were dissolved in hexane at the concentration of 0.1 M. 

### 4.3. RNA Isolation and cDNA Synthesis

Total RNA was isolated using Trizol reagent (Invitrogen, Carlsbad, CA, USA), quantified on a NanoDrop-2000 spectrophotometer (NanoDrop Technologies, Inc., Wilmington, DE, USA) and digested with DNaseI (Fermentas, Glen Burnie, MD, USA) to remove trace amounts of genomic DNA, before synthesis of single-strand cDNA using Revert Aid First Strand cDNA Synthesis Kit (Fermentas). The cDNA of antennae was used as the template for gene cloning and, together with cDNAs from heads (without antennae), thoraxes, abdomens and legs as templates for qRT-PCR.

### 4.4. Gene Cloning and Sequence Analysis

To clone the full-length ORF of *AlucOR46* specific primers were designed using Primer Premier 5.0 software (PREMIER Biosoft International, Palo Alto, CA, USA); their sequences are reported in Table S2. PCR reaction mixtures of 25 μL contained 1 μL cDNA, 0.25 μL primeSTAR HS DNA polymerase, 5 μL 5× PrimerSTAR Buffer, 2 μL dNTP mixture (2.5 mM each) and 0.5 μL of each primer (10 μM). PCR conditions were: initial denaturation at 95 °C for 3 min; 35 cycles of 95 °C for 30 s, 55 °C for 30 s, and 72 °C for 2 min; final extension at 72 °C for 10 min. The amplification product was purified from 1.0% agarose gels and ligated into the pEasy-T3 vector (TransGenBiotech, Beijing, China) following the manufacturer’s instructions. Plasmids were extracted and sequenced at BGI (Beijing, China).

The amino acid sequences of AlucOR12, AlucOR18, AlucOR28, AlucOR30 and AlucOrco were obtained from GenBank (accession numbers: KP010358, KP010359, KP010360, KP010361 and KC881255, respectively). Amino acid sequences were aligned using ClustalW2 [36]. TMDs were predicted using TMHMM Server Version 2.0 [37].

### 4.5. Quantitative Real-Time PCR 

To evaluate the expression of *AlucOR46* in different tissues of male and female *A. lucorum*, qRT-PCR was performed using cDNA from antennae (A), heads without antennae (H), thoraxes (T), abdomens (Ab) and legs (L) on the ABI Prism 7500 Fast Detection System (Applied Biosystems, Carlsbad, CA, USA). To correct for samples variation and normalize *AlucOR46* expression level, *AlucActin* gene (KU188517) was used as a reference. Primers were designed using the Beacon Designer 7.90 software (PREMIER Biosoft International) (Table S2). qRT-PCR reactions were conducted in 20 μL reaction mixtures containing 0.5 μL of each primer (10 μM), 1 μL of sample cDNA, 8 μL of sterilized H_2_O and 10 μL 2× Go Taq qPCR Master Mix (Promega, Madison, WI, USA). The qRT-PCR cycling program was: 95 °C for 2 min, 40 cycles of 95 °C for 30 s, 60 °C for 1 min. Relative quantification was performed by using the comparative 2^−∆∆*C*t^ method, where ∆*C*_t_ = (*C*_t_, OR gene − *C*_t_, reference gene), ∆∆*C*_t_ = (∆*C*_t_, different samples − ∆*C*_t_ maximum). Each experiment was repeated three times using three independently isolated RNA samples.

### 4.6. Vector Construction and cRNAs Synthesis 

The full ORF of *AlucOR46* was amplified by primers with restriction enzyme sites (*Apa*I and *Not*I) (Table S2) and cloned into pT7Ts vector. The vector was linearized by the restriction enzyme *Sma*I and complimentary ribonucleic acid (cRNA) was synthesized from the linearized plasmid using mMESSAGE mMACHINE T7 kit (Ambion, Austin, TX, USA).

### 4.7. Two-Electrode Voltage Clamp Electrophysiological Recordings

Oocytes expression and electrophysiological recording were performed as described in previous reports [24,38]. Mature healthy *Xenopus* oocytes were treated with 2 mg/mL collagenase in washing buffer (96 mM NaCl, 2 mM KCl, 5 mM MgCl_2_, and 5 mM HEPES, pH 7.6) for 1–2 h at room temperature. Then, the *Xenopus* oocytes were microinjected with a mixture of 27.6 ng *AlucOR46* cRNA and 27.6 ng *AlucOrco* cRNA. After injection, oocytes were cultured at 18 °C for 3–7 days in 1X Ringer’s solution supplemented with 5% dialyzed horse serum, 50 μg/mL, tetracycline 100 μg/mL streptomycin and 550 μg/mL sodium pyruvate. Whole-cell currents were recorded from injected oocytes in response to different odors using two-electrode voltage clamp and dose-response curves were obtained at a holding potential of −80 mV. Micropipettes filled with 3 M KCl were used as electrodes. During the recording, oocytes were challenged with a panel of 65 compounds in a random order at a flow rate of 8 mL/min for 15 s. Before next stimulus, 1× Ringer’s solution was used to wash oocyte at a flow rate of 10 mL/min to allow the current to return to baseline. Data acquisition and analysis were performed with Digidata 1440A and pClamp 10.0 software (Axon Instruments Inc., Union City, CA, USA). Dose-response data were analyzed using GraphPad Prism 5.

### 4.8. Electroantennogram Experiment

EAG recordings were used to measure the responses of females and males *A. lucorum* to six plant volatiles, (*S*)-(−)-Limonene, (*R*)-(+)-Limonene, (*E*)-2-Hexenal, (*E*)-3-Hexenol, 1-Heptanol and (*1R*)-(−)-Myrtenol. EAG signals were recorded and analyzed using Syntech IDAC 4 and GC-EAD softwares (Syntech, Hilversum, The Netherlands). Chemicals were dissolved in hexane to the final concentration of 0.1 M and 20 μL of the solutions were applied to a piece of folded filter paper (0.5 cm × 2 cm), which was inserted into a glass Pasteur pipette. The antennae of *A. lucorum* were excised at the base, and few segments were removed at the distal end. The antennae were connected between two glass electrodes filled with 0.1 M KCl using electrode gel. To deliver stimuli, a constant airstream of 30 mL/s produced by a stimulus controller (CS-55, Syntech) was sent through the glass Pasteur pipette for 0.2 s at 10 mL/s airflow. EAG signals recorded from the antenna were amplified by 10× AC/DC headstage preamplifier (Syntech), then acquired by Intelligent Data Acquisition Controller (IDAC-4, Syntech), sent to the computer and finally recorded by Syntech software. Blank and reference stimuli were 10 μL of pure hexane and 10 μL of 0.1 M 1-Hexanol, respectively [29]. Antennae were stimulated with the six compounds in random order. EAG responses for each compound were recorded from six females and six males. 

Relative EAG responses for each compound were calculated by the formula: Relative EAG response = (EAG response to the test compound − mean EAG response to the blank stimulus)/(mean EAG response to the reference stimulus − mean EAG response to the blank stimulus) [39]. The differences of mean relative EAG response between female and male to the same test compound were compared using Student’s *t*-tests. Statistical analyses of the above data were processed in SPSS 23.0.

## Figures and Tables

**Table 1:** The structure of six active compounds and the half maximal effective concentration (EC_50_). values of four compounds.

Odorants	Structure	EC_50_ (M)
(*R*)-(+)-Limonene	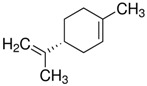	-
(*S*)-(−)-Limonene	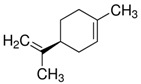	-
(*E*)-2-Hexenal	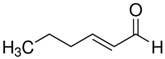	3.71 × 10^−5^
(*E*)-3-Hexenol	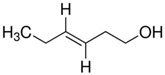	2.53 × 10^−5^
1-Heptanol	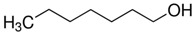	1.38 × 10^−5^
(*1R*)-(−)-Myrtenol	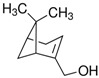	7.44 × 10^−5^

## Supplementary Materials

Supplementary materials can be found at http://www.mdpi.com/1422-0067/17/8/1165/s1.
