# Supplementary material for: A Female-Biased Odorant Receptor from Apolygus lucorum (Meyer-Dür) Tuned to Some Plant Odors
Source: Int J Mol Sci. 2016 Jul 28;17(8):1165. doi: 10.3390/ijms17081165 (PMC5000588; doi:10.3390/ijms17081165)
Supplement: Supplementary file 1 [file ijms-17-01165-s001.pdf]

Supplementary Materials: A Female-Biased Odorant Receptor from *Apolygus lucorum* (Meyer-Dür) Tuned to Some Plant Odors

Zhixiang Zhang, Meiping Zhang, Shuwei Yan, Guirong Wang and Yang Liu

Table S1. All the 65 compounds tested in this study.

Serial Number	Name	CAS
1	2-Phenylethanol	60–12–8
2	(*Z*)-3-Hexenol	928–96–1
3	β-Citronellol	106–22–9
4	Geraniol	106–24–1
5	(*Z*)-2-Hexenol	928–94–9
6	Benzaldehyde	100–52–7
7	(1*S*)-(−)-Verbenone	1196–01–6
8	1-Hexanol	111–27-3
9	(*S*)-(*Z*)-Verbenol	18881–04–4
10	(*Z*)-3-Hexenyl acetate	3681–71–8
11	3,7-Dimethyl-3-octanol	78–69–3
12	(−)-Borneol	464–45–9
13	(+)-Borneol	464–43–7
14	(1*R*)-(−)-Myrtenol	19894–97–4
15	(−)-(*E*)-Pinocarveol	547–61–5
16	(−)-Linalool	126–91–0
17	Linalool	78–70–6
18	Methyl benzoate	93–58–3
19	Myrcene	123–35–3
20	3-Vinylbenzaldehyde	19955–99–8
21	α-Pinene	80–56–8
22	(−)-β-Pinene	18172–67–3
23	Camphene	79–92–5
24	α-Humulene	6753–98–6
25	(*E*)-2-Hexenyl acetate	2497–18–9
26	α-Terpinene	99–86–5
27	(−)-(*E*)-Caryophyllene	87-44-5
28	(−)-Caryophyllene oxide	1139–30–6
29	Heptanal	111–71–7
30	4-Ethylbenzaldehyde	4748–78–1
31	(*R*)-(+)-Limonene	5989–27–5
32	(*E*)-2-Hexenal	6728–26–3
33	1-Heptanol	111–70–6
34	(1*R*)-(−)-Myrtenal	18486-69-6
35	(*E*)-3-Hexenol	928-97-2
36	(*S*)-(−)-Limonene	5989-54-8
37	1,4-Diethylbenzene	105–05–5
38	4′-Ethylacetophenone	937–30–4
39	Cinnamaldehyde	104–55–2
40	(±)-Citronellal	106–23–0
41	2-Undecanone	112–12–9
42	Benzyl alcohol	100–51–6

Table S1. *Cont.*

Serial Number	Name	CAS
44	Hexyl acetate	142–92–7
45	Octyl butyrate	110–39–4
46	Benzyl acetate	140–11–4
47	Tetradecane	629–59–4
48	Nonyl acetate	143–13–5
49	Hexyl acetate	142–92–7
50	Tridecane	629–50–5
51	Methyl salicylate	68917–75–9
52	(±)-Camphor	76–22–2
53	Ocimene	13877–91–3
54	Nerolidol	40716–66–3
55	2,6-Di-tert-butylphenol	128–39–2
56	1-Aminoanthracene	610–49–1
57	2-Pentadecanone	2345–28–0
58	Acetophenone	98–86–2
59	Cumene	98–82–8
60	Cedrol	77–53–2
61	Octyl acetate	112–14–1
62	Ethyl butyrate	105–54–4
63	Ethyl Hexanoate	123–66–0
64	β-Ionone	79–77–6
65	Phenyl benzoate	93–99–2

Table S2. Primers for full length cloning, vector construction and qRT-PCR.

Primer Name	Sequence (5′–3′)
full length cloned	
AlucOR46-F	ATGGGCTACAGAGTTTATCCTCAG
AlucOR46-R	CTATGATACTTCTGCTTCATCTTGAAC
AlucOrco-F	ATGCAGAAAGTGAAGATGCACG
AlucOrco-R	TTATTTGAGCTGCACCAACACC
vector construction	
AlucOR46-F	tcagggcccgccaccATGGGCTACAGAGTTTATCCTCAG
AlucOR46-R	tcagcggccgcCTATGATACTTCTGCTTCATCTTGAAC
AlucOrco-F	tcagggcccgccaccATGCAGAAAGTGAAGATGCACG
AlucOrco-R	tcagcggccgcTTATTTGAGCTGCACCAACACC
qRT-PCR	
AlucOR46-F	GCTACCTCTTCACATCCATCTAC
AlucOR46-R	CAGCAACGGTGTGCTTATTG
AlucActin-F	CCTTCCTGGGTATGGAATCTTG
AlucActin-R	TGTTGGCGTACAGGTCTTTC
